# GrooVR: an open access virtual reality drumming application to improve pro-sociality using synchronous movement

**DOI:** 10.3389/fpsyg.2025.1536761

**Published:** 2025-04-28

**Authors:** Liam Cross, Wesley Nixon, Jack Smith, Chia-huei Tseng, Yoshifumi Kitamura, Isamu Endo, Juris Savostijanovs, Gray Atherton

**Affiliations:** ^1^School of Psychology, University of Plymouth, Plymouth, United Kingdom; ^2^Department of Psychology, Institute of Population Health, University of Liverpool, Liverpool, United Kingdom; ^3^Research Institute of Electrical Communication, Tohoku University, Sendai, Japan; ^4^Interdisciplinary ICT Research Center for Cyber and Real Spaces, Tohoku University, Sendai, Japan; ^5^Independent Researcher, Greater Manchester, United Kingdom

**Keywords:** synchrony, coordination, virtual reality, pro-sociality, cohesion, cooperation, prejudice, joint action

## Abstract

Interpersonal synchrony can enhance social bonding, cooperation, and reduce negative biases, especially toward out-group members. However, studying social synchrony faces practical challenges. To address this, we introduce a customizable virtual reality (VR) application and report two experiments evaluating its effectiveness. In the first experiment, participants drummed either in sync or out of sync with a virtual partner matching their gender, age, and ethnicity. Synchronous drumming increased feelings of affiliation but did not influence pro-social behavior in an economic game. The second experiment involved Caucasian participants drumming with Middle Eastern avatars. Synchronous drumming not only increased trust and affiliation but also reduced prejudicial attitudes toward Middle Eastern refugees. These findings suggest that virtual synchrony can strengthen social bonds and decrease bias, offering both theoretical insights and practical applications.

## Introduction

### Synchrony and its pro-social effects

Synchrony can be defined as two or more entities coordinating their movements over time to a common rhythm (Phillips-Silver et al., 2010); examples can be seen in the way planets move in lockstep or the flashing displays of fireflies falling into rhythm with their neighbors (Strogatz, 2003). We also engage in these synchronous acts and displays, matching the gait of those we walk with or chanting at sports, political or religious events (McNeill, 1997). Engaging in synchrony has been shown to have widely cascading pro-social consequences amongst those who take part, including increasing affiliation or rapport (Hove and Risen 2009), pro-social attitudes (Atherton et al., 2019), helping (Cross et al., 2020) and cooperation in economic games (Wiltermuth and Heath, 2009). For example, synchronously drumming with people has been shown to increase how likely and for how long people are willing to spend helping their co-actors in lab-based tasks (Kokal et al., 2011) and increases cooperation between children (Kirschner and Tomesallo, 2009).

Synchrony has also been shown to help inter-group relations (Cross et al., 2019; Good et al., 2017; Webb et al., 2017). In one study, Atherton et al. (2019) asked Hungarian participants to walk in silence for 2 min with a member of the Roma community. Those who had done so synchronously demonstrated greater affiliation and reduced implicit and explicit prejudice to not only the individual they had walked with but the wider social group, as compared to those who walked asynchronously. Similar effects of synchrony have also been demonstrated in the UK towards Middle Eastern refugees (Atherton and Cross, 2020). The social effects of synchrony have now also been found to occur and sustain outside of the lab (Pearce et al., 2016) for at least 24 h following instances of coordination (Cross et al., 2020).

### Problems in the field

Despite a growing body of literature demonstrating these effects (for recent reviews see Cross et al., 2019; Mogan et al., 2017; Rennung and Göritz, 2016; Vicaria and Dickens, 2016), this field of enquiry has suffered from criticisms common to much of social psychology concerning replication issues and expectancy effects (Atwood et al., 2022). Furthermore, the field is also hampered by various pragmatic barriers to movement and group research, which may partly explain some diverging findings.

Firstly, there is an unfortunate trade-off often made between ecological validity and experimental control and measurement in ones choice of synchrony tasks. For example, taking the two most prominent examples of synchrony, creating pro-sociality in tasks mimicking real-life examples of synchrony, like cup waving and singing (Wiltermuth and Heath, 2009), offer very little experimental control with no quantifiable measurement of synchrony reported. More tightly controlled, quantifiable measures of finger tapping (Hove and Risen 2009) are not very ecologically valid, immersive or engaging. Furthermore, ensuring the relevant type and degree of coordination between participants can itself present significant challenges. It can be difficult to experimentally achieve the required synchrony between two people due to individual differences that may inhibit a person from being capable of synchronizing (Carnevali et al., 2024; Georgescu et al., 2020; Leroy et al., 2015).

The impact of participant demographics has also been shown to have myriad effects on both manipulation and measures. For example, the nationality and personality of participants have been shown to affect the synchrony/pro-sociality relationship (Cross et al., 2019). Miles et al. (2010) demonstrated that participants coordinate less with individuals who arrive just a few minutes late for a study. Some studies have relied on confederate partner paradigms (Atherton et al., 2019; Cross et al., 2020; Hove and Risen 2009; Kokal et al., 2011) or mentally simulated synchrony (Atherton et al., 2019; Cross et al., 2017, 2020; Crossey et al., 2021) to solve some of these issues, however these solutions raise issues of their own relating to the loss of control and measurement with imagined paradigms and confederate cost, performance and reliability.

### Virtual synchrony

Virtual Reality (VR) may offer an elegant and cost-effective alternative that can afford solutions to many of the issues the field faces as was recently suggested by Tunçgenç et al. (2023). It offers a customizable, immersive yet controllable and measurable environment in which all aspects of both agent and environment can be manipulated as required. Virtual partners can be easily set to reliably coordinate with participants to the required degree and their aesthetics quickly amended as wished.

In this paper, we detail a customized VR drumming application built to study synchrony and its social consequences where various relevant parameters can be instantly changed using user-friendly Graphical User Interfaces. This open-access application allows researchers to efficiently utilize this application with no knowledge of computer languages, VR or the Unity engine. It offers a rich and stimulating environment to study synchrony, where the relevant parameters (such as environment, partner aesthetics, movement type, (i.e., leader-follower vs. joint goal) degree of synchrony, etc.) can all be easily manipulated at a click. It offers precise control of environment and partner behavior and built-in recordings of relevant measures while also being an immersive and engaging user experience for participants. This open-access VR environment, GrooVR, is described, here along with two experiments testing its utility to replicate the pro-social consequences of synchrony.

### The current paper

Here we detail two experiments designed to evaluate whether our VR application GrooVR is capable of fostering similar pro-social affects as have been shown to follow actual and imagined synchrony. In Experiment 1 we replicate two of the most common pro-social consequences that have been shown to follow physical and imagined synchrony self-report ratings of affiliation (Cross et al., 2017; Hove and Risen 2009; Lang et al., 2017; Pearce et al., 2017; Tarr et al., 2016;) and only physical synchrony—behavioral measures of pro-sociality in economic games (Cross et al., 2016, 2020; Reddish et al., 2013; Wiltermuth and Heath, 2009). Experiment 2 then explores whether virtual synchrony can affect affiliation, behavior and attitudes towards out-group members as has been shown to follow both physical and imagined synchrony (Atherton et al., 2019; Cross et al., 2021).

## Experiment 1 methods

### Materials—the GrooVR application

**Figure 1:**
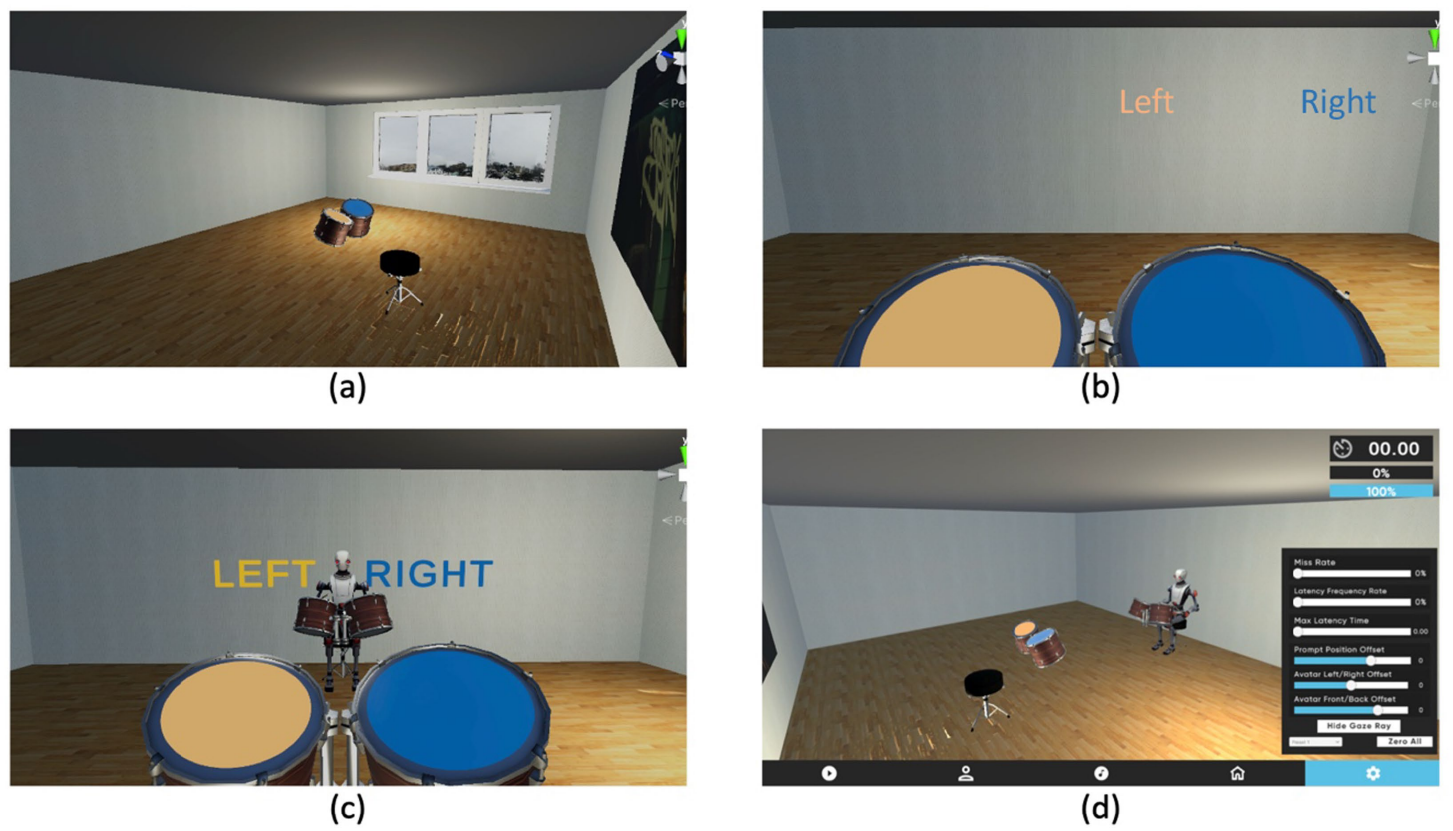
The virtual reality environment. **(a)** The initial virtual environment, **(b)** the participant’s point of view in the environment. **(c)** The participants’ point of view in the environment once an avatar has been loaded in. **(d)** The experimenter view, showing the adjustment panel for avatar positioning and synchrony adjustment.

GrooVR was created in the Unity game engine and is compatible across all current popular VR headsets, Oculus rift, Valve Index, HTC Vive models, Meta Quest 2 onwards. The oculus rift S was used here. The virtual environment consists of a square room with white walls, wooden flooring, and a single window (see Figure 1), designed to closely mirror the lab space in which participants took part. Within the room is a drum stool where the participant is positioned, and two drums colored yellow (left) and blue (right) are directly in front of them, in an easy-to-reach distance. A virtual partner can then be loaded at a desired position in front of the participant, and a virtual metronome can be displayed beyond them.

Several properties can be easily adjusted by the experimenter in real-time using sliders, including the position of the partner avatar, drums and the visual metronome, as well as whether the metronome is omnipresent, absent, or only present for an adjustable guiding period. The avatar’s appearance, choice of backing track and environment can all be easily adjusted, as well as the avatar’s movement behavior. This can be adjusted to affect how the avatar synchronizes to either the backing track or the participant (allowing for either leader-follower or joint goal paradigms) as well as the degree of success it exhibits in doing so (see Figure 1d). This is done using 3 metrics: Missed Hit Frequency, Late Hit Frequency and Maximum Latency.

**‘Missed Hit Frequency’** refers to the probability that the partner avatar misses a beat in the drumming rhythm, playing either nothing or the incorrect drum (i.e., the right drum when it should be the left). This is measured from 0 to 100% chance of occurrence and is calculated on each beat, meaning a value of 50% signifies a 50% chance any given beat will be missed, rather than 50% of beats in the track are missed. The **‘Late Hit Frequency’** property works in the same way as the **‘Missed Drum Hit Frequency’** property; however, it alters the chance that any given note is delayed and played out of time rather than skipped entirely, working in conjunction with the final property: **‘Maximum Latency’**, which affects to what extent any particular note can be delayed, with a value range of 0 to 1 beat relative to the beats per minute of the selected track, meaning at most a note can be played a full beat after it should have been, although the latency is always a random value.

Two coordination measures (Error Rate and Synchrony Rate) are updated and displayed live to the experimenter during the session on an external display (and are output in a. CSV file). The ***Error Rate*** refers to the participant’s success in matching the drumming pattern dictated by the metronome. This is calculated as the time difference between when a note should be played and when it was played, either early or late. This is then represented as a percentage, with 100% being perfectly on time and 0% as entirely out of time, with the threshold being the next beat in the sequence, i.e., the most out of synch a note could be if it were not to be hit until right before the next beat in the sequence, or immediately after the previous hit. The ***Synchrony Rate*** shows the synchrony between the participant and the avatar and is calculated as follows: the differences in time between participant and partner avatar drum hits are measured; if these are below a threshold of 0.2 s (as was used in Atherton et al., 2019), the hit is said to be in synchrony and awarded a score of 100%, if a hit is missed then a score of 0% is allocated, and if the hit is not missed but also not within the perfect synchrony threshold, a percentage score is calculated based on how close to perfect synchrony each hit was.

In both experiments reported here, the avatar was placed at a comfortable distance in front of the participant, with the metronome appearing to the left and right of the avatar for the entire duration of the trial, as seen in Figure 1c. The coordination type involved both the participant and the avatar following the beat with various degrees of accuracy. The avatar parameters in the synchrony condition were set to 5% for Missed Hit Frequency, 5% for Late Hit Frequency and 0.1 for Maximum Latency. These parameters ensured the avatar performed the task with around 95% accuracy in terms of perfect synchrony to the beat, therefore allowing participants who performed close to the beat to be in synchrony with the avatar. In the non-synchronous condition, the adjustable parameters were set to 80% for Missed Hit Frequency and late Hit Frequency and 1 for Maximum Latency. These parameters ensured the avatar performed the task incorrectly and unpredictably, making it difficult for participants to achieve synchrony with the avatar.

### Procedure

**Figure 2:**
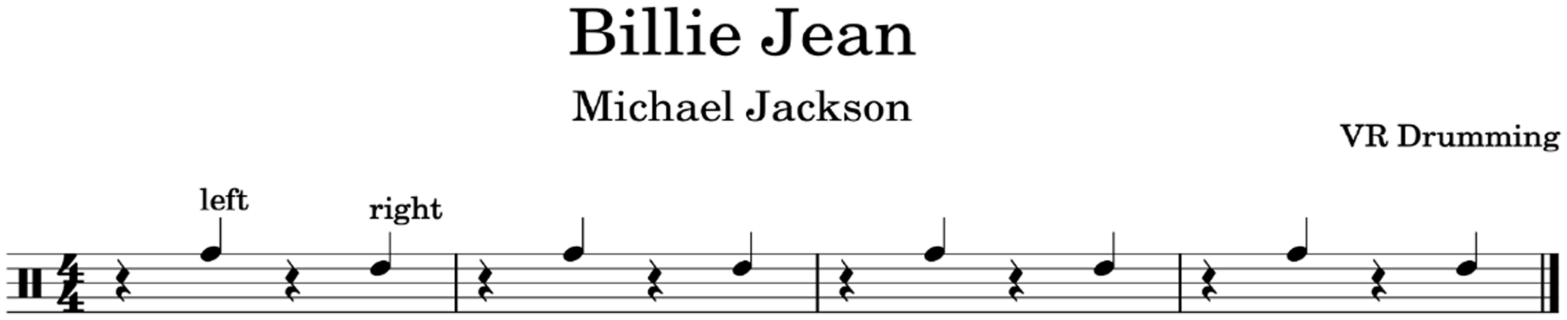
Rhythmic pattern of Billie Jean drumming beat.

Upon entering the lab, participants were seated, acclimatized into the VR environment, and asked to look around and familiarize themselves with the scene. They then practiced free drumming (without any accompanying music or partner) to become accustomed to the feel and positioning of the virtual drums. Once the participants felt comfortable, a backing track began playing along with a visual metronome indicating when to hit each drum that appeared directly above the relevant drums (see Figure 1c). For Expt 1. this was an instrumental (no lyrics) version of Billie Jean by Michael Jackson (see Figure 2 for the drumming pattern). The song choice, speed, and drumming pattern were chosen due to the strong beat and ease of acquisition for novices during piloting. Pilot data suggested most participants could easily drum along with the required beat to around 80% accuracy after a few minutes of practice.

**Figure 3:**
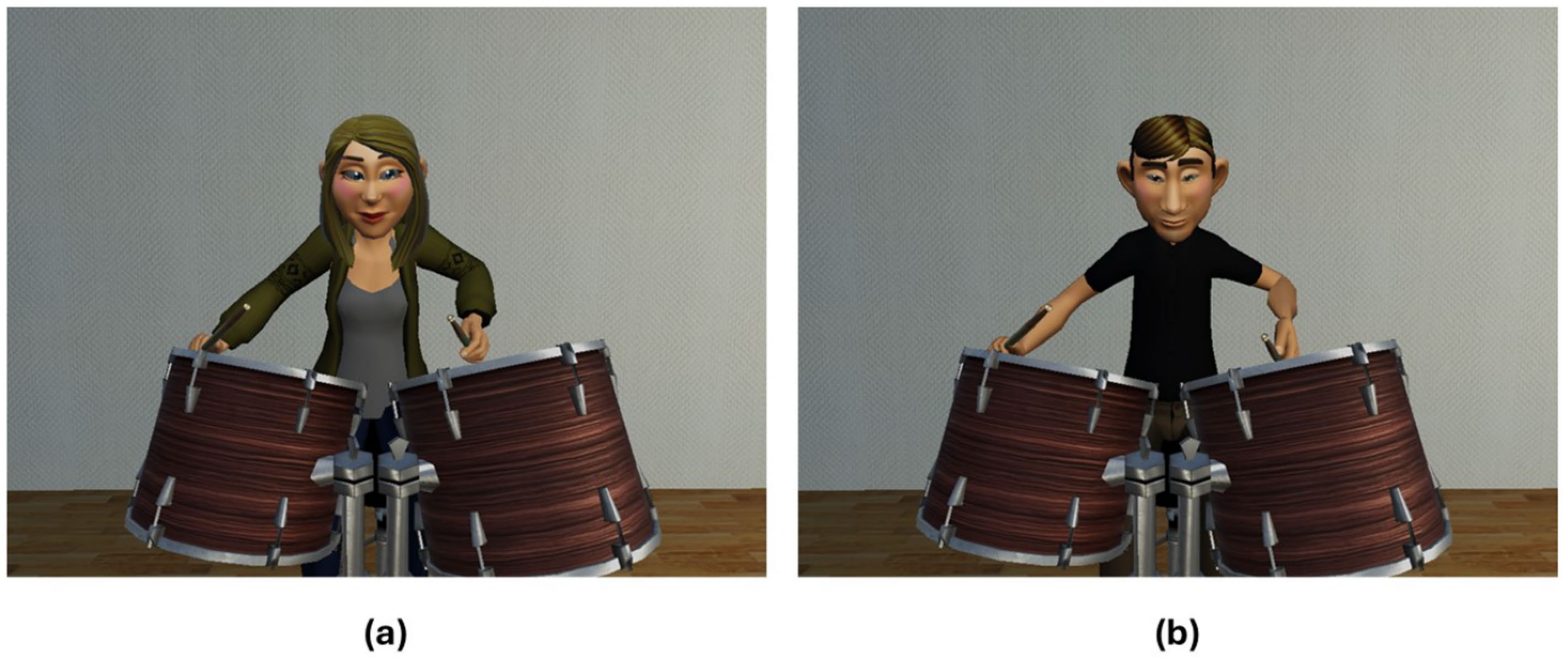
Caucasian adult avatars. **(a)** Female, **(b)** male.

Participants spent approximately 2 min (range 1–3 min) practicing playing along until they had learnt the relevant rhythmic drumming sequence (performing above 80% accuracy, as indicated on the external experimenter display, but unseen to participants). All participants reached 80% accuracy within this training period. Once participants had completed this practice period, a gender and ethnicity-matched Caucasian partner avatar appeared directly in front of them in the virtual environment (see Figure 3). Participants who identified as non-binary were asked to choose the avatar that matched most closely with their preferred gender (the two participants who identified as non-binary chose to drum with the female avatar).

Participants in both conditions (Synchronous and Non-Synchronous) were instructed to follow the drum prompts (metronome) regardless of what the avatar did (condition). Once participants had completed 3 min of drumming, they were asked to complete measures of affiliation and pro-sociality in that order.

### Measures

#### Affiliation

The affiliation measure was taken from Cross et al. (2016, 2017) and consisted of four questions: How connected do you feel to the avatar you drummed with? How much would you like to see the avatar you drummed with again? How much would you say you were on the same team as the avatar you drummed with? How well do you feel you understood the avatar you drummed with? Participants answered each question on a 5-point Likert scale ranging from not at all (1) to very much so (5). **This measure has previously been shown to have good internal consistency** (Cross et al., 2016, 2017).

#### Pro-sociality

Participants then played a two-round donation economic game measuring altruism and trust as has been previously used by Glaeser et al. (2000). In the first round, measuring altruism, the participants played the role of the allocator, with the virtual partner being the recipient. Participants were provided with £10 and were asked to decide how much of the £10 (from 0–10 in integers of 1) they wished to donate to their virtual partner. In the second round of measuring trust, the roles were reversed, and participants took the role of the recipient. They were told the virtual partner had also allocated an amount to them and they could (before knowing that amount) decide whether to take the allocated amount or a pre-set amount of £4.50.

### Design, participants and ethics

This experiment employed a between groups design with one independent variable (movement type – Synchronous or Non Synchronous) and three dependent variables (Affiliation, Altruism and Trust). Eighty Caucasian individuals participated in this study (25 males, 53 females, 2 other, *M*_age_ = 20.63 yr., *SD*_age_ = 3.72). Participants were recruited via opportunity and snowball sampling and semi-randomly assigned to a Synchronous or Non-Synchronous condition (keeping gender and total Ns equal across conditions). The only inclusion criteria were that participants were Caucasian and at least 18 years old. All participants were naïve to the aims of the study. This study took approximately 20 min to complete, and participants were compensated £3 or SONA credits for their time. The experiment was approved by the ethics board of the Psychology department at Edge Hill University.

## Experiment 1 results

**Figure 4:**
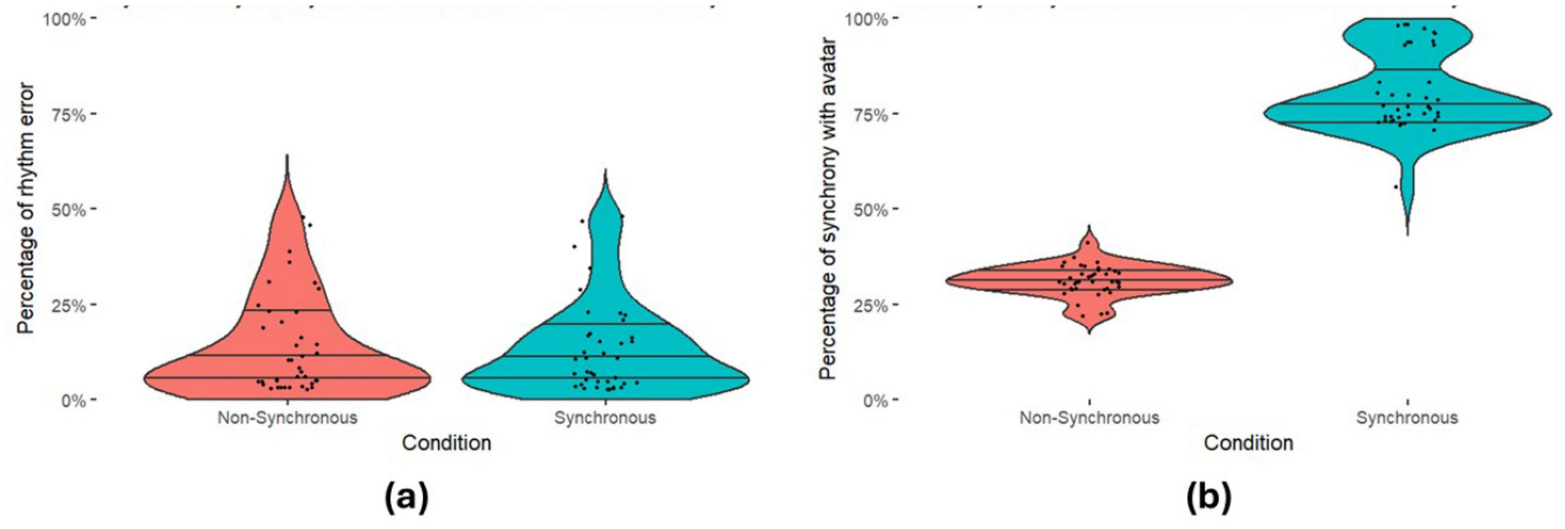
Participant drumming performance. Violin plots illustrate the distribution of **(a)** Error Rates (with beat) **(b)** Synchrony Rates (with the avatar). The width of the violins reflects the data density. Median lines and quantile markers (25th, 50th, and 75th percentiles) provide insights into central tendencies and variability.

All the data was first checked for normality using Shapiro Wilks tests. Where distributions significantly differed from normal non**-**parametric tests were used. We then checked whether participants were performing the drumming task as instructed There was no significant difference in error rates (how well participants matched the required rhythm) between those in the Synchronous and Non-Synchronous conditions [*U* = 777.50, *p* = 0.828], suggesting participants in both conditions were equally able to keep to the specified rhythm. Next, we checked that the percentage of synchrony with the avatar was greater in the Synchronous condition than in the Non-Synchronous condition. Those in the Synchronous condition were significantly more synchronous with the avatar than those in the Non-Synchronous condition, [*U* = 0.00, *p* < 0.001, *R^2^* = −0.742]. See Figure 4 for descriptives for these two measures. We finally checked that all participants performed their task adequately by looking to see if any participants fell above or below expected synchrony thresholds in their respective condition (which was predetermined to be >50% synchrony in the Synchrony condition and <50% synchrony in the Non-Synchronous condition), which no participants did. These results indicate that our virtual reality drumming task was suitable for participants to perform adequately, and our manipulation of synchrony created the desired context for us to interpret the following results.

**Figure 5:**
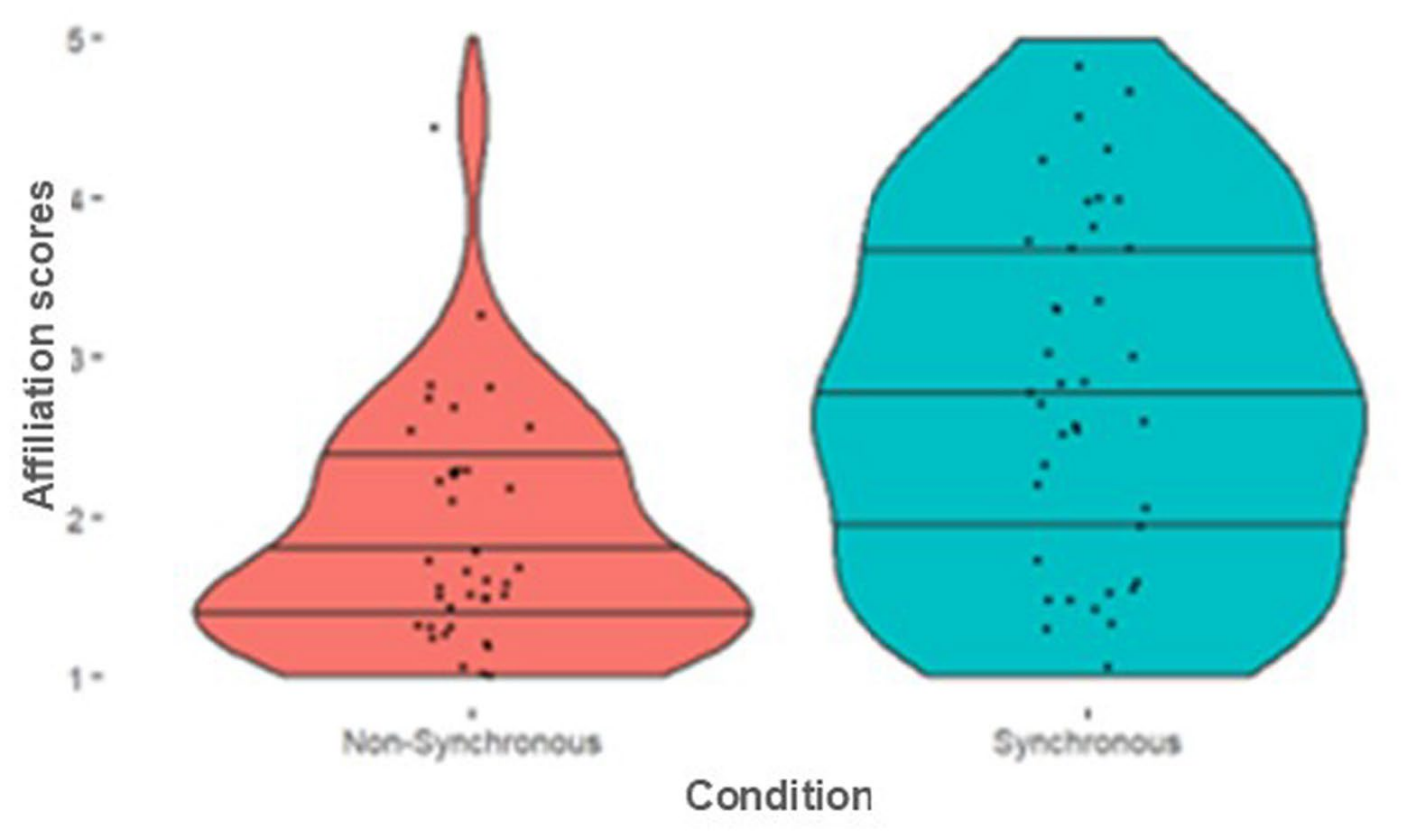
Violin plots illustrate the distribution of Affiliation scores between movement conditions.

We combined the four affiliation items into a single composite score by taking the mean Cronbach’s alpha confirmed internal validity, a = 0.840. As predicted, those in the Synchronous condition reported significantly greater affiliation with the virtual partner than those in the Non-Synchronous condition, [*U* = 409.00, *p* < 0.001, *R^2^* = 0.179]. See Figure 5 for descriptives. A simple linear regression also showed that synchrony rates significantly predicted affiliation scores (*R^2^* = 0.156, *F*(1, 78) = 14.469, *p <* 0.001), while error rates did not (*R^2^* = 0.002, *F*(1, 78) = 0.159, *p* = 0.691).

Lastly, we explored whether there was a difference in the behavioral measures of pro-sociality between conditions. While those in the Synchronous condition (*M* = 4.08, SD = 2.09) did donate more to the partner than those in the Non-Synchronous condition (*M* = 3.48, SD = 2.01) on average, this difference was not significant [*U* = 684.00, *p* = 0.228]. We also conducted a simple linear regression to test if synchrony scores significantly predicted the amount donated, which they did not (*R^2^* = 0.013, *F*(1, 78) = 1.06, *p* = 0.306). We then explored whether there was a difference between conditions for round 2 (trust). A chi-square test of independence showed there was no relationship between the choices of those in either condition for round 2 of the economic game (trust), [*χ^2^* (1, 80) = 0.464, *p* = 0.496], see Table 1 for descriptives. Again, a binary logistic regression showed that Synchrony rates did not predict choices in round 2 of the economic game [*χ2* (1) = 0.189, *p* = 0.664].

**Table 1:** Descriptive statistics for round 2 of the economic game in Experiment 1.

		Pre-set donation	Avatar donation	Total
Condition	Synchronous	15	25	40
	Non-synchronous	18	22	40
Total		33	47	80

## Experiment 1 discussion

Experiment 1 showed that participants in both conditions were able to perform the required movement task in VR to an acceptable accuracy and achieved the required level of synchrony with the avatar. Those who drummed synchronously reported greater affiliation towards that avatar than those who drummed non-synchronously with them, and synchrony rates predicted affiliation, but error rates did not. Neither altruism nor trust in the economic game differed between conditions, nor were these variables predicted by synchrony rates.

This disparity between self-report (affiliation) and behavioral (altruism/trust) measures could be explained in a few different ways,

1. it may be that it does not make sense to ask participants to play an economic game with an avatar; these games are typically played with real individuals who have a utility case for the relevant resources being distributed (money), which an avatar does not. That said, research has shown that synchronous movement with an avatar can influence subsequent economic games (Fujiwara et al., 2021), though the effects may be lessened compared to when playing with humans.
2. it may be that virtual synchrony acts like imagined synchrony and affects self-report measures of attitudes but not behavior. Cross et al. (2017) for example found that mentally simulated synchrony affects self-report measures of affiliation, but not behavioral pro-sociality in economic games. It may be the case that virtual synchrony’s effects are closer to imagined than actual synchrony.
3. it may be that group dynamics mediate the synchrony pro-social behavior relationship. A great deal of work has now shown that the effects of synchrony are larger and more stable when people are coordinating with out-group members, or in some cases are not present at all when people coordinate with in-group members. Equally some work has shown that people construe their identities in more de-individuated and interpersonal ways post coordination, and this mediates some of the pro-social effects of synchrony (Atherton et al., 2019; Cross et al., 2019, 2020; Crossey et al., 2021; Miles et al., 2011; Good et al., 2017; Tunçgenç and Cohen, 2016). That is, the mechanism for synchrony to affect behavior is that it leads individuals to view their coordinated co-actors as common group members. Since participants drummed with age, gender, and race-matched avatar, this social categorization may not have occurred here.

We further explored these eventualities in Experiment 2 where we replicated Experiment 1 to disentangle which of the possible explanations may best explain the pattern of findings. This time, rather than taking part with an avatar representing a member of one’s ethnic group, participants were paired with an out-group avatar with a Middle Eastern appearance. This group was chosen as this is a prominent out-group in the UK and has been successfully used in the UK population with imagined synchrony paradigms (Atherton and Cross, 2020). In line with this work, the partner avatar was referred to as a Middle Eastern refugee. As well as disentangling this pattern of findings from Experiment 1, this study aimed to replicate work on imagined synchrony, showing a reduction in prejudicial attitudes towards out-groups with virtual synchrony.

## Experiment 2 methods

Eighty Caucasian individuals participated in this study (19 males, 58 females, one other, *M*_age_ = 19.95 yr., *SD*_age_ = 4.95). The sampling method, study time, compensation and inclusion criteria were identical to Experiment 1, with one additional exclusion criteria being participants who had taken part in Experiment 1. This experiment was also approved by the ethics board of the Psychology department at Edge Hill University. Experiment Two’s materials and procedures were identical to those in Experiment 1, bar the following additions/amendments.

**Figure 6:**
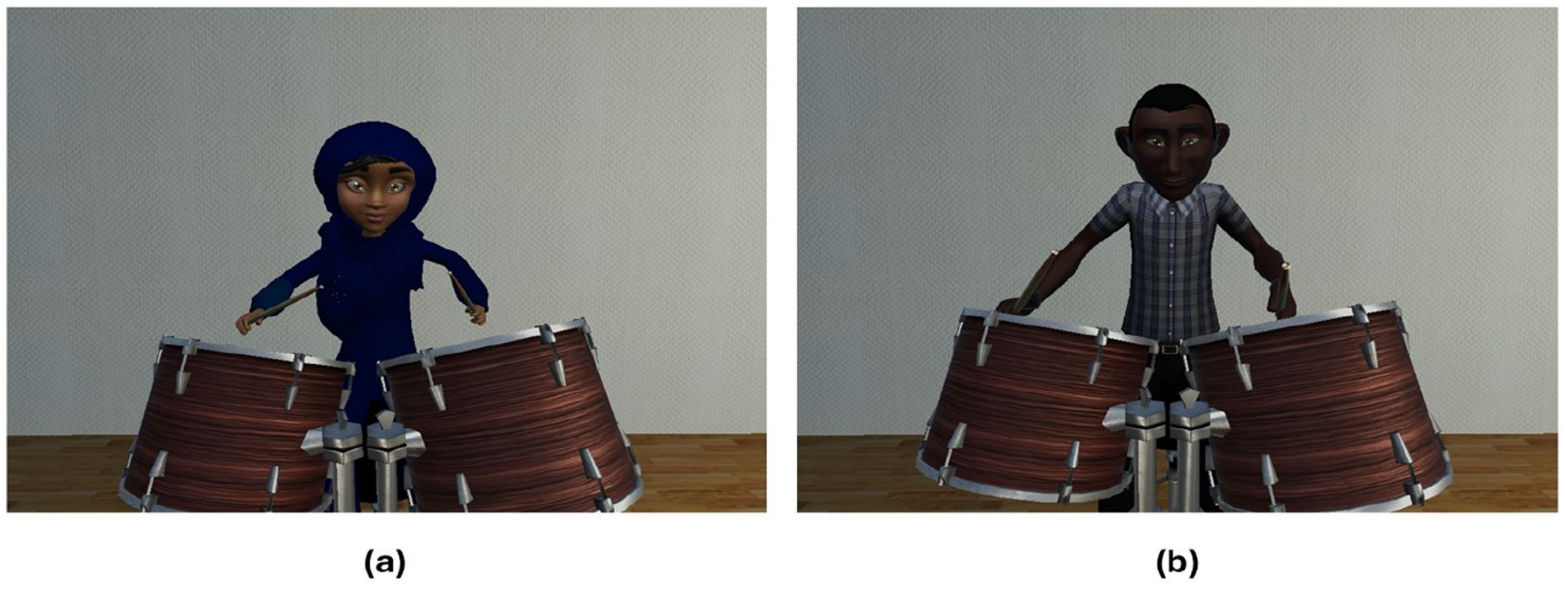
Middle eastern avatars. **(a)** Female middle eastern avatar, **(b)** male middle eastern avatar, used in Experiment 2.

**Figure 7:**
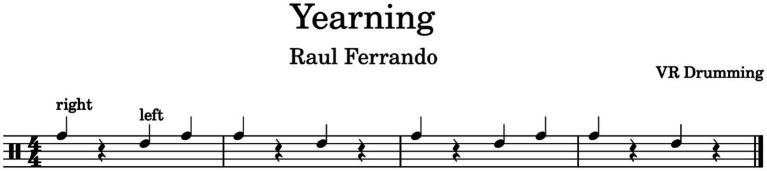
Rhythmic patterns of Yearning drumming beat.

In Experiment 2 participants drummed along with a gender and age-matched Middle Eastern partner avatar (see Figure 6, The participant who identified as ‘other’ chose to drum with a female avatar), to an instrumental (no lyrics) version of Yearning by Raul Ferrando (see Figure 7). This song was chosen for its Middle Eastern sound through its use of string instruments and traditional percussion instruments, while also retaining a simple and steady drum beat. Pilot data again indicated people could easily play along to around 80% accuracy after a few minutes of practice.

In addition to the measures taken in Experiment 1, participants also completed a measure of prejudicial attitudes after the drumming manipulation, affiliation measure and economic game. The prejudice measure consisted of three questions (taken from Atherton et al., 2019; Cross et al., 2020 which also showed good internal validity for this measure). They included the questions Would you consider Middle Eastern refugees to be aggressive? Would you be happy to have a Middle Eastern refugee as your boss? Do you feel Middle Eastern refugees are given too much government support? Participants answered each question on a 5-point Likert scale from not at all (1) to very much so (5). Question 2 was reverse-coded so that a larger score indicated greater prejudice.

## Experiment 2 results

**Figure 8:**
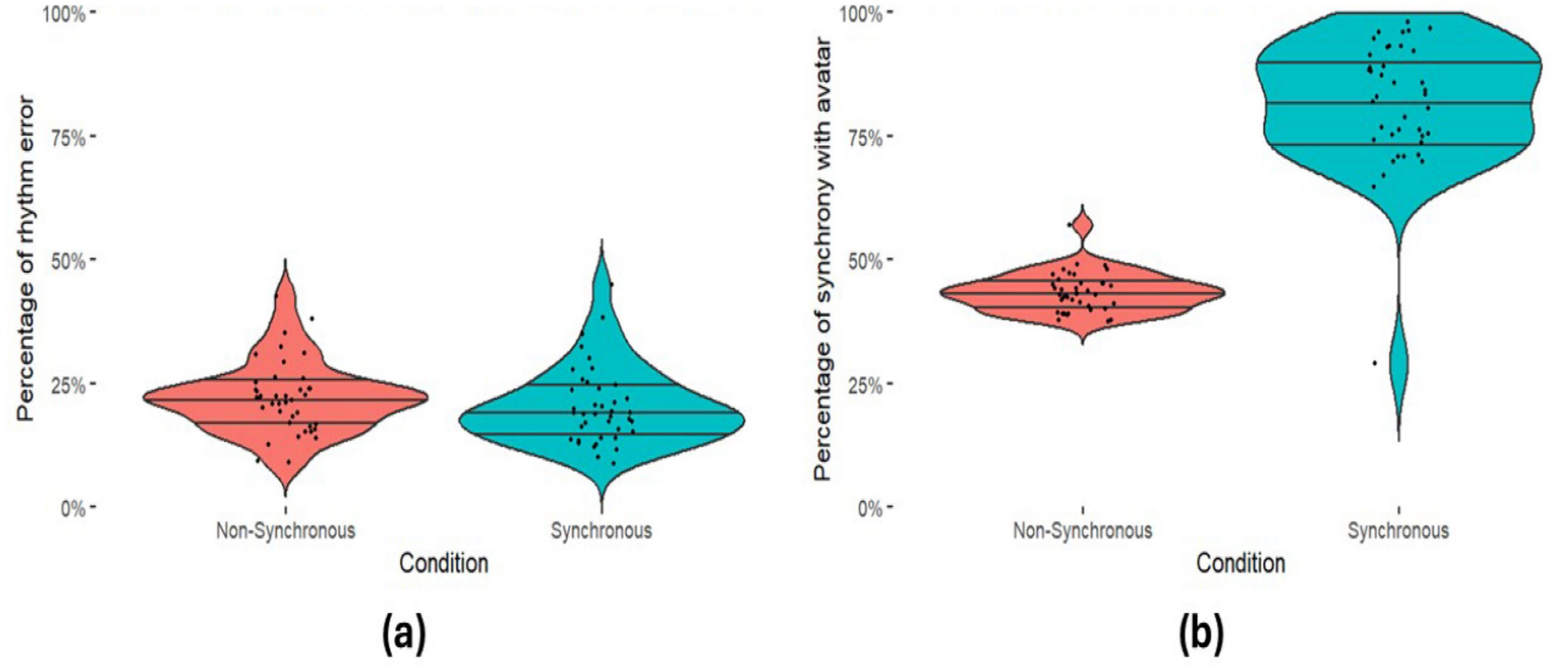
Participant drumming performance. Violin plots illustrate the distribution of **(a)** Error Rates (with beat) **(b)** Synchrony Rates (with the agent).

We first checked whether participants in each condition were performing the task adequately as we did in Experiment 1. Participants in both conditions performed similarly in terms of their Error Rate [*U* = 651.00, *p* = 0.15], and Synchrony Rates in the Synchrony condition were significantly greater than the Non-Synchronous condition [*U* = 40.00, *p* = <0.001, *R^2^* = −0.669]. See Figure 8 for descriptives for both of these measures. We then checked that all participants achieved an adequate percentage of synchrony with their virtual partner relative to their condition. Two participants (one from each condition: a participant in the Synchronous condition achieving 29% synchrony and the participant in the Non-Synchronous condition achieving 57% synchrony) had their data removed from further analysis as they surpassed the 50% prerequisites.

**Figure 9:**
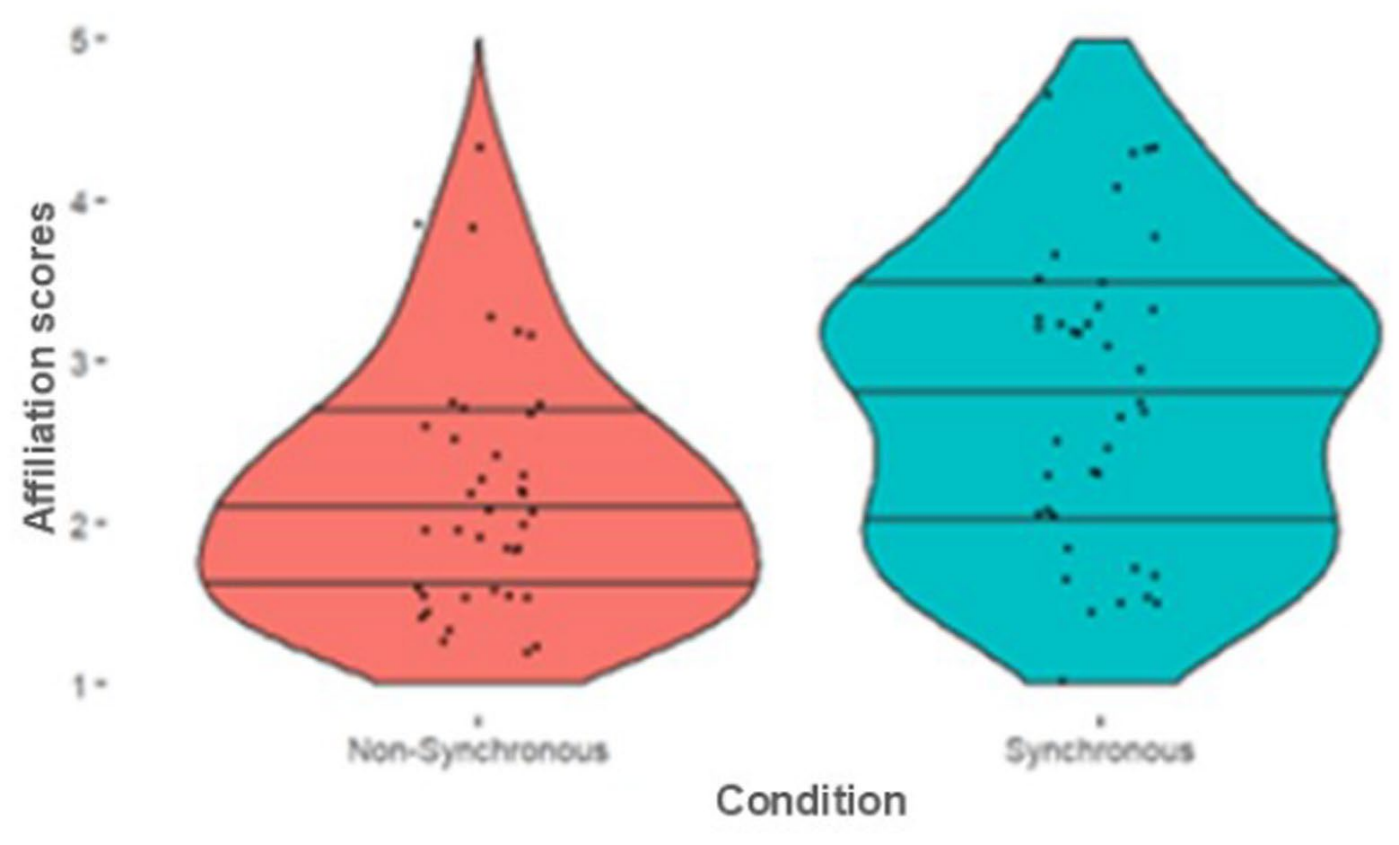
Affiliation scores between groups, violin plots illustrate the distribution of scores.

We then explored whether there was a difference in affiliation ratings, Cronbach’s alpha confirmed internal validity a = 0.789. Those in the Synchronous condition again reported greater affiliation with the virtual partner than those in the Non-Synchronous condition [*U* = 482.00, *p* = 0.005, *R^2^* = 0.100]. Please see Figure 9 for descriptives. Again, a simple linear regression also showed that Synchrony Rates significantly predicted affiliation scores [*R^2^* = 0.169, *F*(1, 76) = 15.49, *p* < 0.001], while Error Rates did not (*R^2^* = 0.015, *F*(1, 76) = 1.141, *p* = 0.289).

**Figure 10:**
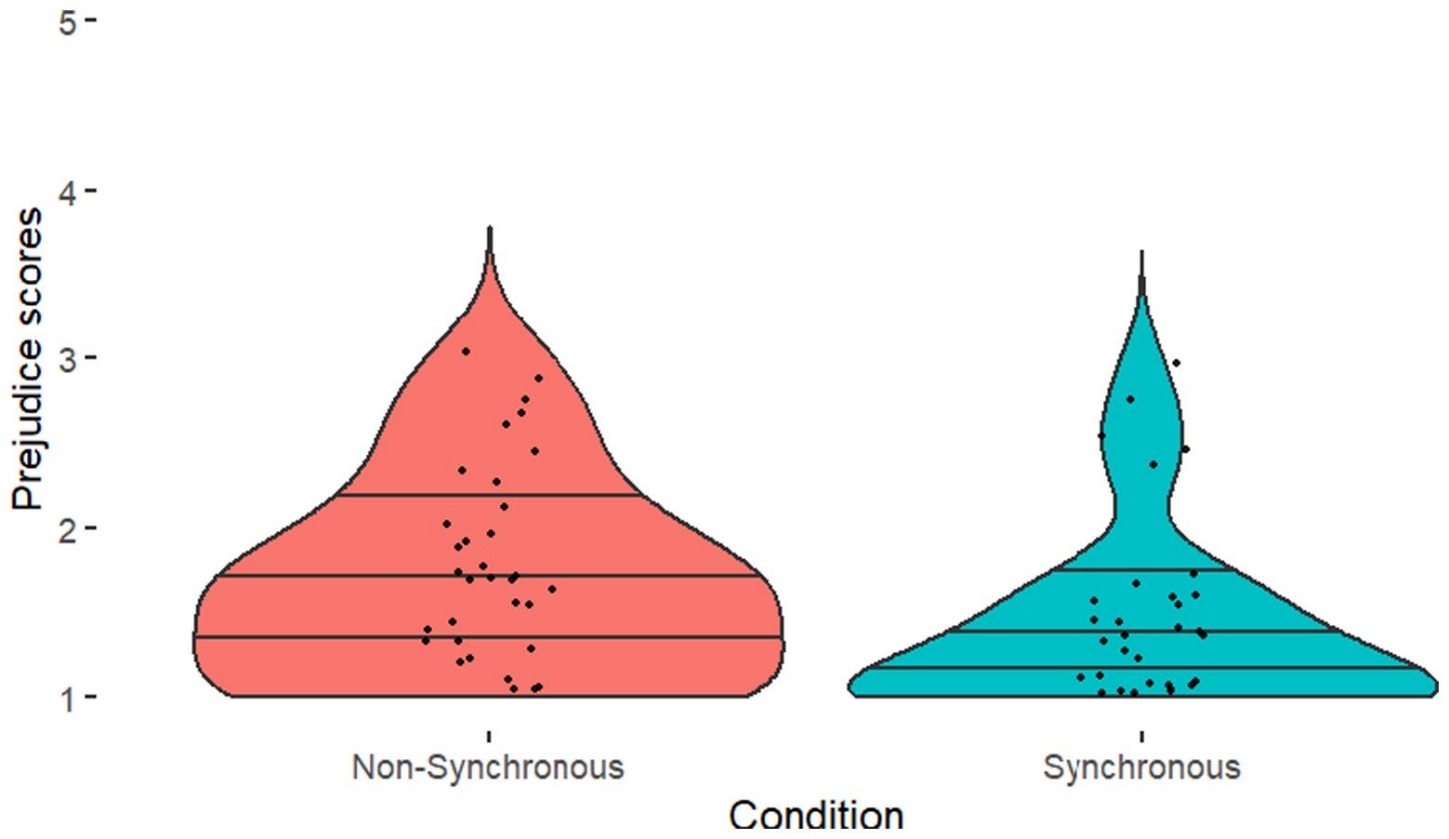
Prejudice scores between groups, violin plots illustrate the distribution of prejudice scores.

We then explored whether there was a difference between conditions in ratings of prejudicial attitudes combined into a composite score, Cronbach’s alpha confirmed internal validity a = 0.670. As predicted, those in the Synchronous condition reported significantly less prejudicial attitudes compared to those in the Non-Synchronous condition [*U* = 519.50, *p* = 0.013, *R*^2^ = 0.080]; see Figure 10 for the descriptives. We also conducted a simple linear regression to test if Synchrony Rates significantly predicted prejudicial attitudes, which it did not (*R^2^* = 0.044, *F*(1, 76) = 3.494, *p* = 0.065), nor did Error Rates (*R^2^* = 0.001, *F*(1, 76) = 0.073, *p* = 0.788).

There were no significant differences in the amount donated in round 1 of the economic game between those in the Synchronous condition (*M* = 5.08, SD = 1.86) and the Non-Synchronous condition (*M* = 5.26, SD = 1.67), [*U* = 719.00, *p* = 0.641]. A simple linear regression showed Synchrony Rates did not significantly predict the amount donated (*R*^2^ < 0.001, *F*(1, 76) < 0.001, *p* = 0.983). However, a chi-square test of independence did reveal a significant association showing those in the Synchronous condition were more likely to take the avatar’s donation and those in the Non-Synchronous condition were more likely to take the pre-set amount [*χ^2^* (1, 78) = 4.768, *p* = 0.029]. See Table 2 for descriptives. We also conducted a binary logistic regression to ascertain if Synchrony Rates could predict this choice, which they did [*χ^2^* (1) = 4.255, *p* = 0.039].

**Table 2:** Descriptive statistics for round 2 of the economic game in Experiment 2.

		Pre-set donation	Avatar donation	Total
Condition	Synchronous	8	31	39
	Nonsynchronous	17	22	39
Total		25	53	78

## General discussion

In this paper, across two studies, we tested the social effects of synchronous movements with avatars in virtual environments. In Experiment 1, we tested whether individuals who drummed with gender, age and ethnicity-matched avatars (synchronously or not) showed differences in pro-sociality, including affiliation, altruism and trust. We found greater self-reported affiliation towards the avatars when participants drummed Synchronously with them, which is in line with previous research. We did not, however, find any differences in the behavioral measures of pro-sociality using an economic game. Given the theory that the social effects of synchrony are most stable and pronounced when coordinating with a member of a different social group, as synchrony serves as a form of social ‘glue’ that can cross social boundaries (Cross et al., 2019), we next tested whether virtual synchrony with an out-group partner could elicit greater behavioral pro-sociality in participants.

In Experiment 2, we had participants complete the same tasks, only this time, they drummed with an avatar depicting a middle eastern refugee. In line with experiment 1, we showed that participants could perform the required movement task to an acceptable accuracy level and that those in the relevant conditions achieved the required level of synchrony. Again, those who drummed Synchronously with an out-group avatar reported greater affiliation towards that avatar than those who drummed Non-Synchronously with them, and synchrony rates again predicted affiliation. This time, we also took self-report measures of prejudicial attitudes towards Middle Eastern refugees. Those in the Synchronous condition reported significantly lower prejudicial attitudes compared to those in the Non-Synchronous condition, though these were not significantly predicted by Synchrony Rates.

Moving on to the behavioral measures, while altruism in the economic game did not differ between conditions nor was predicted by Synchrony Rates, trust was affected by synchrony. Trust was significantly associated with condition (more trust amongst those who drummed in synchrony) and predicted by Synchrony Rates. Research suggests that trust and synchrony have a bidirectional effect on one another. Mitkidis et al. (2015) found that during economic trust games, participants synchronize heart rates, indicating an autonomic response in people to couple with a person with whom they hope to build trust. Launay et al. (2013) found that people instructed to synchronize with a metronome also exhibited more trust. This is in line with a broader area of work suggesting that synchronization may make people more cooperative and more susceptible to conformity (Wiltermuth and Heath, 2009), with many real-life examples such as responding to orders in army ranks or following religious guidelines, both being organizations that regularly use synchronous movements in their practices and have shown to have particularly high rates of conformity (Deng et al., 2023; Thiruchselvam et al., 2017). Interestingly, we did not find that altruism in the economic game was increased in the synchronous condition. Other research has found that following synchrony, participants are more altruistic towards a confederate whom they perceive as being a victim (Valdesolo and DeSteno, 2011). However, Fujiwara et al. (2021) found that synchrony led to higher altruism only in settings where the outcome was a ‘win-win.’ In ‘win-lose’ negotiations, synchrony did not affect altruism. This is in line with our results, which only tested ‘win-lose’ settings (i.e., giving more to the other results in getting less for the self), and similarly did not find an effect of synchrony on altruism.

This pattern of findings provides evidence that virtual synchrony can replicate the effects of actual and imagined synchrony. Although not directly tested, Experiment 2’s finding also sheds some light on the three posited explanations from Experiment 1’s pattern of findings, suggesting that explanation 3 (that group dynamics mediate this effect) is more likely than 1 (economic games are not appropriate or sensitive measures for use with for virtual partner avatars) and 2 (virtual like imagined synchrony only affects attitude and not behavior). As seen in a similar study by Atherton et al. (2019), it appears that just as in real-life synchrony and imagined synchrony, virtual synchrony with out-group members reduces prejudice and leads to greater pro-sociality towards that individual and their wider group.

## Limitations

There are some limitations to note. Firstly it is important to note that we only tested Caucasian participants and their feelings and behavior towards either Caucasian or Middle Eastern agents. We do not know if these findings would necessarily generalize in the other direction, if synchrony would lead minority group participants to change their thoughts, feeling, behavior, or attitudes towards majority groups, Equally we note that our sample did not have an equal gender representation, there may also be other confounding factors such as personal music preferences, VR experience, or individual differences in rhythm perception /ability and baseline attitudes that would all likely be present in a more complete evaluation of virtual synchrony’s relationship with pro-sociality. We were unable to capture and account for all of these variables here. Future work may wish to do so. Lastly it is worth noting that some findings here, did not reach significance (altruism and trust in Experiment 1, and altruism in Experiment 2), although sample sizes here are larger than many comparable sample sizes in the field (for a review see Cross et al., 2019). We cannot rule out that this was due to insufficient power, though this is unlikely as self report measures of affiliation tend to show smaller effect sizes than behavioral ones form economic games (Mogan et al., 2017). To explore this, postdoc power analysis was performed using G power, for an effect size of 0.3 (Mogan et al., 2017) a sample of 80 affords >75% power for a Mann Whitney *U* test.

## Implications, applications and future directions

The social effects of synchrony have been used purposefully, or appeared as an artifact, in rituals as old as humanity itself (McNeill, 1997). Without a doubt, humans today interact with one another in ways that would have been inconceivable to our ancestors engaging in an ancient drumming circle hundreds of thousands of years ago. Despite these advances, our research shows the timelessness of synchrony (Gelfand et al., 2020). Our results suggest that virtual environments hold the potential to change the landscape of psychological research. It can change the landscape of virtual interventions, shaping through VR interactions the way in which people have real life interactions. What these studies show, more than anything, is that synchrony’s effects are not lost when elicited through a digital environment.

As discussed earlier in this paper, psychological research on synchrony, and more broadly, in-group/out-group effects, are suggested to suffer from experimenter effects and pragmatic difficulties, which VR may offer avenues to ameliorate. Indeed, VR allows for the study of synchrony between groups without these constraints. Furthermore, psychological research itself is often confined to a lab. The effects it finds, which may benefit the general public, are difficult to replicate in everyday life. The digitalization of communication and media has led to widespread changes in the accessibility and application of psychological research. For instance, digitizing mental health care has led to increased use of online therapy (Comer, 2015), including using online avatars to improve the client/counsellor relationship (Rehm et al., 2016). There are digital games for improving understanding and improving mental health (Lau et al., 2017) and cognitive functioning (Starks, 2014). VR is arguably the next frontier in this process, with the ability to fully immerse an individual in a different world.

While people (especially those from relevant out-groups such as different cultures) are not able to easily be physically co-present, through VR, they can representationally inhabit the same space and experience joint movement as if they were together. As this research shows, virtual coordination alone carries significant psychological effects. It changes how a person feels about a different agent and, importantly, as shown in Experiment 2, how they feel about different social groups. Reducing prejudice through virtual interactions is one of the ways in which lab-based experiments can be quite easily distributed in the wider community. In this way, this work not only answers questions about the feasibility of synchrony research in virtual environments but also shows a path more broadly towards creating research that can have real-world impact. Research shows that it is possible to reduce prejudice through intervention (Hsieh et al., 2022; Lemmer and Wagner, 2015). The strength in this paradigm lies in not only the unique, non-verbal method for prejudice reduction, but also offers an open platform solution that will bridge the gap between the lab and real life.

There are several future directions for this work. First, the accessibility of this work means that it can be used in various settings and populations. For instance, this work has the potential to be used in therapeutic and rehabilitation settings, where VR interventions have been shown to improve coordination in people with movement differences such as improving outcomes for people with Parkinson’s Disease (Dockx et al., 2016) and stroke patients (Laver et al., 2017). Our drumming paradigm also importantly shows the potential to reduce prejudice and in-group bias. This could be used in community settings where there is strong intergroup conflict, and indeed VR is an emerging area of prejudice reduction due to its immersion and high degree of flexibility (Chen and White, 2024).

Our experiment hinges on the notion that participants interact with agents that they believe represent real life constructs. While virtual realities inspire feelings of immersion and a suspension of reality, participants in our studies were not made to believe that they were interacting with real people and, indeed, were explicitly told that they were interacting with avatars. There is, however, scope to have two real participants interact through VR, independent of their locations. The knowledge that you are, for instance, interacting with the avatar of a real-life Middle-Eastern refugee, who in turn is interacting with your avatar, all in real-time, may enhance the effects found in Experiment 2. Future research may want to explore how to further this application to include real-life VR encounters, which, while perhaps leading to lower synchrony levels (as the avatar in our studies had near-perfect rhythm execution), may add to the perception of out-group contact, which has been shown to decrease prejudice in and of itself (Pettigrew and Tropp, 2006). Because VR allows people to inhabit the same space vicariously, it may be a powerful way to have members from particularly polarized groups (groups in active conflict, groups with significant stigma and persecution) coordinate with members of the majority group who may not ordinarily have exposure to real-life people in these groups. Likewise, this may be a safe way for people in groups that may be at risk of real-life violence or persecution to engage with members of the majority, thus improving intergroup relations.

## GrooVR–revisited

**Figure 11:**
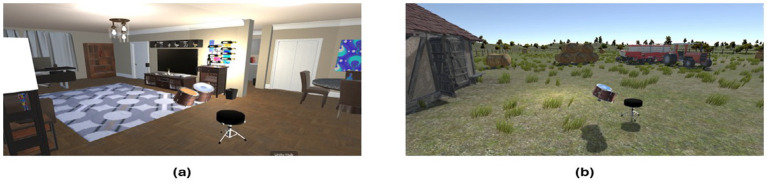
Example of various environments available to be selected. Environment **(a)** is built to depict a typical sitting room in the UK, which could be used to match the Caucasian avatar. Environment **(b)** is built to depict a typical farm environment, which could be used to match with the non-human avatar.

**Figure 12:**
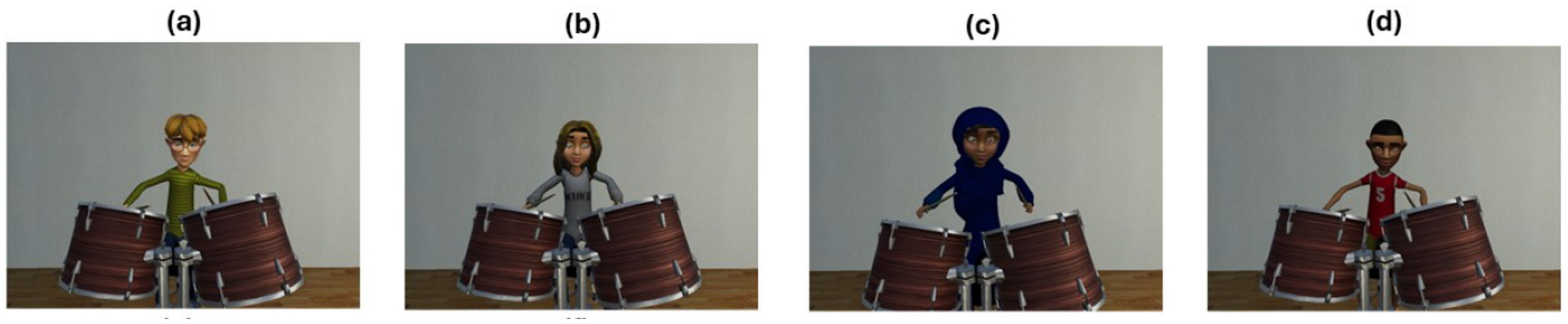
Child avatars, Caucasian male **(a)**, female **(b)** and middle eastern female **(c)** and male **(d)**.

The VR application detailed throughout this paper is freely available to interested parties for research purposes and can be found at https://www.neuroplaylab.com/measures-and-code. Finally, we detail below a couple of extra functionalities that have been added to the application since data was collected, namely, eye tracking and some more child-oriented additions. Four additional avatars representing Caucasian and Middle Eastern children have been added, along with a choice of virtual environments, an apartment or a Middle Eastern market offering a more immersive context for the relevant backing tracks and avatar types. (Please see Figures 11, 12 for illustrative examples of these various choices).

**Figure 13:**
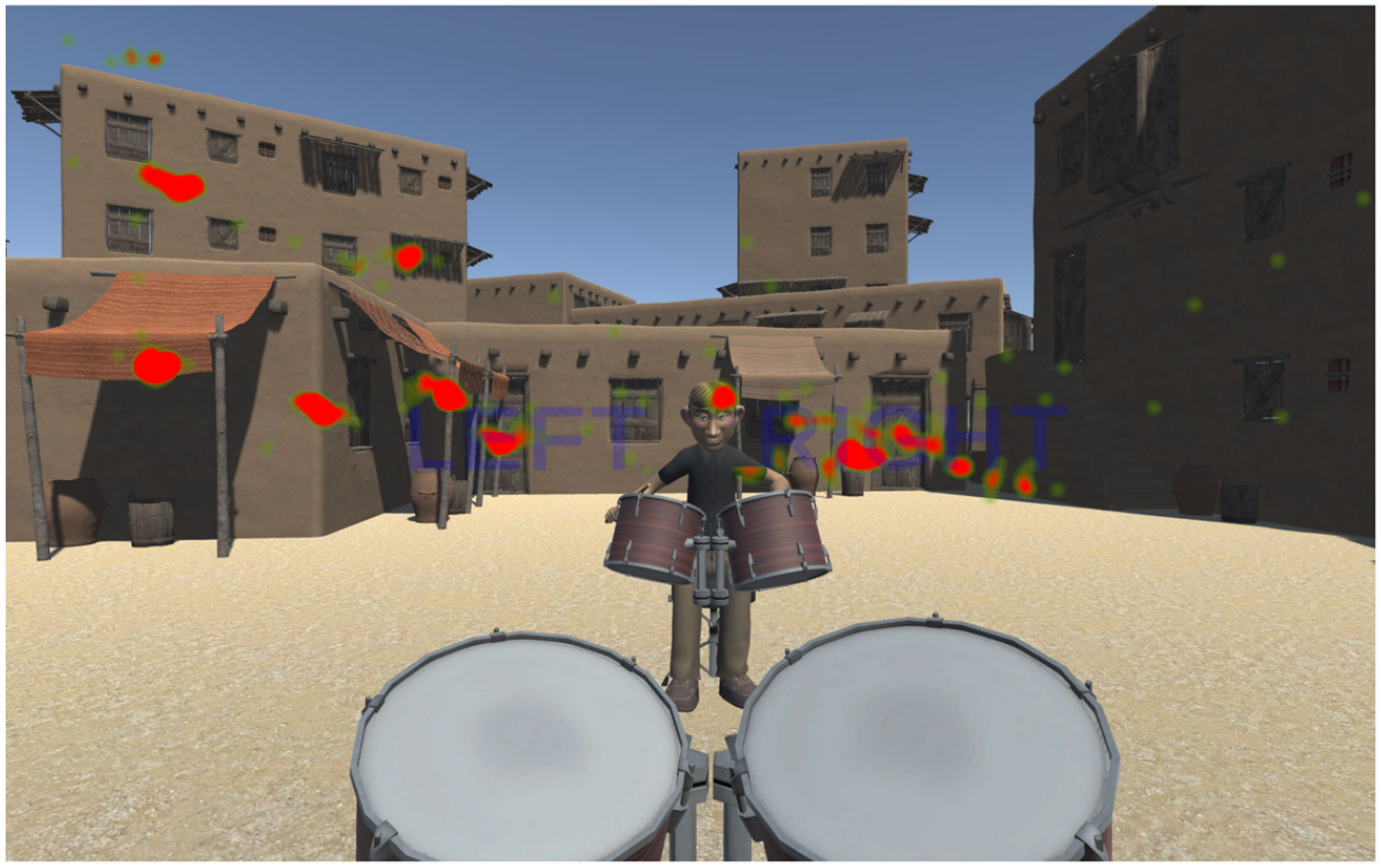
Example of ‘Heatmap Viewer’ application that allows the visualization of eye-tracking data, with green highlighting areas that were looked at briefly, and red showing areas that were looked at most frequently.

Furthermore, eye-tracking functionality has now also been incorporated into HTC Vive Eye Pro headset as well as the oculus’s eye racking modules. This makes use of the SR Runtime application (version 2.1.25.5) provided by HTC, along with the SRanipal Unity plugin (version 1.3.6.8) that works in conjunction with SteamVR. Eye tracking works by collecting the focal point of a participant’s gaze during each frame update and then outputting relevant information into a CSV file for each scene used. Relevant information includes the x, y, and z coordinates at which a participant was currently focused; where possible, a named description of the object currently being looked at by the participant (e.g., partner avatar’s head, left drum, right metronome prompt, etc.); a timestamp at which the gaze was recorded, so that gaze times for individual objects could be retroactively calculated if desired. In conjunction with the CSV output, a further application was developed to visualize the eye-tracking data from the CSV file in a 3D environment that can be freely explored and match the scenes from the experiment. An example is provided in Figure 13, highlighting areas with the most significant number of gaze points in red and those with fewer gazes in green.

## Data Availability

The raw data supporting the conclusions of this article will be made available by the authors, without undue reservation.
